# Development and Validation of a Measure for Seeking Health Information in the Diabetes Online Community: Mixed Methods Study

**DOI:** 10.2196/55424

**Published:** 2024-07-04

**Authors:** Allyson S Hughes, Sarah Beach, Spruhaa Vasistha, Nazanin Heydarian, Osvaldo Morera

**Affiliations:** 1 Department of Primary Care Ohio University Heritage College of Osteopathic Institution Athens, OH United States; 2 Denison University Granville, OH United States; 3 School of Social Work University of Texas at Rio Grande Valley Edinburg, TX United States; 4 Department of Psychology University of Texas at El Paso El Paso, TX United States

**Keywords:** online health information, health information seeking, digital health, digital technology, digital intervention, social support, social media, diabetes distress, diabetes, type 2 diabetes, type 1 diabetes, scale development, chronic disease, telehealth

## Abstract

**Background:**

Individuals with chronic diseases often search for health information online. The Diabetes Online Community (DOC) is an active community with members who exchange health information; however, few studies have examined health information brokering in the DOC.

**Objective:**

The aim of this study was to develop and validate the Attitudes Toward Seeking Health Information Online (ATSHIO) scale in a sample of adults with type 1 diabetes (T1D).

**Methods:**

People with T1D were recruited through the DOC, specifically Facebook and Twitter. They were provided with a Qualtrics link to complete the survey. This was a mixed methods study that used thematic analysis along with existing theory and formative research to design the quantitative ATSHIO scale.

**Results:**

A total of 166 people with T1D participated in this study. Confirmatory factor analyses determined a 2-factor scale (*Trusting and Evaluating Online Health Information in the DOC* and *Engaging With Online Health Information in the DOC*) with good convergent validity and discriminant validity. Correlations were found between social support, online health information–seeking, diabetes distress, and disease management.

**Conclusions:**

The ATSHIO scale can be used to investigate how people with diabetes are using the internet for obtaining health information, which is especially relevant in the age of telehealth and Health 2.0.

## Introduction

As health information is readily accessible on the internet, there has been a shift in how individuals with chronic diseases are acquiring information about their condition [1]. People with type 1 diabetes (T1D) typically seek health information online from their peers and share anecdotal evidence and published articles [2]. However, health practices that work extremely well for one person may be ineffective or even detrimental for another person. People with T1D are also encouraged to engage in social support [3], which can exert a positive effect on disease management and is a key factor for psychological adjustment [4], health information–seeking [5], and maintaining mental health [6] and physical health [7,8]. In addition, for individuals with T1D, this social support is often experienced on social media platforms such as Facebook and Twitter/X [9]. More recently, the Diabetes Online Community (DOC) has emerged as a network of individuals with diabetes to engage in discussion on various social media platforms, including Reddit, YouTube [10], Instagram [11], and TikTok [12]. There are many psychosocial benefits to participating in online chronic disease groups such as the DOC [13]. Individuals with diabetes who participate in online support groups report increased empowerment [14], as well as increased positive emotional experiences, positive attitudes toward T1D, and engagement in T1D management behaviors [2].

In this study, we sought to clarify several gaps in the literature due to the nature of existing health information–seeking measures not being tailored to individuals with chronic conditions. In particular, various existing psychological assessment tools do not consider whether an individual has a chronic condition. The Krantz Health Opinion [15], the Miller Behavioral Style scale [16], Threatening Medical Situation [17,18], and the Autonomy Preference Index [19,20] are assessment tools that do not lend themselves to chronic conditions, as these measures propose a hypothetical medical condition and prompt responses based on these hypothetical conditions. Moreover, few studies have been performed in the context of the DOC to collect data on online health information–seeking [13,21].

Health information–seeking is most often studied in three contexts: a hypothetical threatening health situation, behavior change, and prevention. The Krantz Health Opinion [15] focuses on decisions that are actively occurring in a hospital room. The reliability for the item scores ranges from poor to acceptable. The Miller Behavioral Style scale [16] is a widely used measure that assesses coping, specifically monitoring and blunting behaviors. This scale poses four hypothetical threatening situations followed by four monitoring and blunting options for participants to choose from for each provided scenario. This scale has displayed poor to acceptable reliability. Lastly, the Threatening Medical Situation [17,18] measures monitoring and blunting during a medical threat presented using four vignettes (eg, headache, hypertension diagnosis, potential heart surgery, and appendicitis).

Therefore, this study can fill these gaps through the development and validation of a scale that measures seeking health information online for individuals with T1D and examining the relationships between key constructs.

## Methods

### Mixed Methods Framework

This study used a mixed methods approach for scale development [22], involving feedback and inductive and deductive information in a strictly online setting. Items for the developed Attitudes Toward Seeking Online Health Information (ATSHIO) scale were established in previous studies [23-25]. A qualitative pilot study found that participants were using online peer-to-peer–provided health information to decide whether they would seek health care [23]. The scale was then developed based on the pilot study results and a review of the literature. Subsequent studies then focused on investigating the constructs and gaining feedback on the scale [24,25]. Participants provided feedback on the wording of the items; thus, the scale used in this study included the edited and refined items based on this feedback.

### Participants

Participants were eligible for the study if they met the following criteria: (1) 18 years or older, (2) identifying as a member of the DOC, and (3) having been diagnosed with T1D by a doctor. Participants were recruited from the DOC via Facebook posts; tweets using the hashtags #doc, #type1 diabetes, and #dsma; and peer-to-peer referrals.

### Ethical Considerations

This study was approved by the Institutional Review Board at the University of Texas at El Paso (1216875-1). Participants received a US $10 tango gift card upon completing the study.

### Measures

Participants were provided access to a link to the Qualtrics survey where they responded to questions on demographics, a health questionnaire, the eHealth Literacy scale [26], the Social Provisions scale [27], the Treatment Adherence scale [28], and the Diabetes Distress scale [29]. Participants also provided qualitative feedback on the clarity, esthetics, relevancy, tone, and cultural competence of the ATSHIO scale, along with the length of time needed to respond. The scale items are provided in Table 1.

**Table 1:** Items of the Attitudes Toward Seeking Health Information Online scale.

Item number	Item description
1	I frequently use the internet to gain health advice in the Diabetes Online Community.
2	I review multiple internet sources in the Diabetes Online Community before making a health decision for myself.
3	I do not follow the health information that I find on social media in the Diabetes Online Community.^a^
4	I trust the health information that I find in the Diabetes Online Community.
5	I feel comfortable receiving health advice in the Diabetes Online Community.
6	I trust the health information that my friends on social media (Facebook, Twitter, Instagram, discussion forums) provide in the Diabetes Online Community.
7	I feel confident in my knowledge of the available online health resources in the Diabetes Online Community.
8	It is difficult for me to find health information online in the Diabetes Online Community.^a^
9	I feel confident in my ability to find accurate health information in the Diabetes Online Community.
10	When I am confronted with a health problem, I can usually find several solutions via advice in the Diabetes Online Community.
11	I prefer to get advice about medical devices (insulin pumps and CGMs^b^) from the Diabetes Online Community instead of my doctor.
12	When trying to understand my symptoms, my first resource is social media in the Diabetes Online Community.
13	I share health articles on my social media account(s) in the Diabetes Online Community.
14	I do not post health-related items on social media (Facebook, Twitter, Instagram, and/or discussion forums) in the Diabetes Online Community.^a^
15	I prefer to read the health information that I find on social media websites but not engage in online conversations about the health information in the Diabetes Online Community.^a^
16	I feel comfortable providing advice to others in the Diabetes Online Community.

^a^Item is reverse-coded owing to the negative phrasing.

^b^CGM: continuous glucose monitor.

### Data Analysis

Confirmatory factor analysis (CFA) was performed using Mplus 7.11 [30]. Following the suggestions of Brown [31], a variety of plausible models were tested, including a 3-factor model and a 2-factor CFA model, each with 16 items. Robust maximum-likelihood estimation was used in these models. The absolute fit indices included the Satorra-Bentler scaled χ^2^ statistic and the standardized root mean square residual (SRMR). The relative fit indices included the Tucker-Lewis index (TLI) and the comparative fit index (CFI). Following factor analysis and model fit comparison guidelines [32], the CFA results were compared to assess the model fit according to a threshold of SRMR<0.09 in combination with either a TLI or CFI<0.96 or root mean square error of approximation (RMSEA)>0.06.

## Results

### Descriptive Statistics

A total of 175 people with T1D agreed to participate in the study. Nine participants were excluded due to not meeting the inclusion requirements. Of the 166 participants included in this sample, 89.8% (n=149) identified as female with an average age of 34.33 (SD 11.249) years. The majority (149/166, 89.8%) of sample participants were living in the United States. Approximately 86.1% (143/166) of participants identified their race as White. The average household income was US $85,425.28 (median US $74,500). Most participants (133/166, 80%) reported obtaining additional education after high school. The average hemoglobin A_1c_ was 7.3% (SD 1.36%) and more than half of the participants (88/166, 53%) reported using an insulin pump. Of note, 81.9% (136/166) of the participants indicated that they take additional medications beyond insulin. Table 2 summarizes the main demographic and health-related characteristics of the sample.

**Table 2:** Demographic and health-related characteristics of the sample (N=166).

Characteristics		Participants, n (%)
**Race/ethnicity**		
	White	143 (86.1)
	Black/African American	3 (1.8)
	Mexican American	6 (3.6)
	Hispanic or Latino	5 (3)
**Comorbidities**		
	Anxiety	44 (26.5)
	Celiac disease	8 (4.8)
	Depression	55 (33.3)
	Eating disorder	24 (14.3)
	Eye disease	14 (8.4)
	Gastroparesis	11 (6.6)
	Graves disease	6 (3.6)
	Hashimoto disease	12 (7.2)
	Renal disease	3 (1.8)

### Qualitative Assessment of the ATSHIO Scale

Participants provided many detailed responses from questions that should be added to the ATSHIO scale and overall general comments for improvement:

The questions reflect an understanding of what t1s typically do in the online space. One question I would have liked to see, or at least something I’d add, is that my decision to follow advice in the DOC often depends on how well I feel I “know” the person giving the advice. (i.e, is he/she active in DOC, have I interacted with him/her in DOC, etc).

ID 110

Participants were also asked to address the cultural competency of the ATSHIO scale: “Each question was something someone living with type 1 diabetes could answer or relate to” [ID 129]. One participant identified how the items correctly reflected what individuals with T1D experience: “They understood the DOC is able to help through the disease, especially to avoid an appointment with the endo since those are hard to get sometimes” [ID 179]. Participants stated that the survey used participant-endorsed terminology and that questions seemed to indicate that the research team had knowledge of T1D, largely due to the level of detail.

### Reliability of Measures

#### Reliability Based on the Cronbach α Coefficient

The reliability of the quantitative scales was assessed using the Cronbach α coefficient. Every scale exhibited good to excellent reliability (see Table 3).

**Table 3:** Scale and subscale reliability.

Scale	Reliability (Cronbach α)
eHealth literacy	0.897
Social provisions	0.936
Attachment	0.845
Social integration	0.796
Reassurance of worth	0.687
Reliable alliance	0.828
Guidance	0.854
Opportunity for nurturance	0.802
Treatment adherence	0.889
Diabetes distress (T1-DDS^a^)	0.937
Powerlessness	0.820
Management distress	0.760
Hypoglycemia distress	0.860
Negative social perceptions	0.841
Eating distress	0.766
Physician distress	0.883
Friend/family distress	0.860
Attitude toward seeking health information online	0.839
Trusting and evaluating online health information in the DOC^b^	0.789
Engaging with online health information in the DOC	0.746

^a^T1-DDS 1: Type 1 Diabetes Distress Scale.

^b^DOC: Diabetes Online Community.

#### Reliability Based on CFA

##### Three-Factor Model With 16 Items

First, we used CFA to evaluate a 3-factor model with 16 items (see Table 4 for factor loadings). A high correlation was found between factor 1 and factor 2 (*r*=0.942), with moderate correlations found between factor 1 and factor 3 (*r*=0.364) and between factor 2 and factor 3 (*r*=0.492). The following indices did not demonstrate a good model fit: Satorra-Bentler χ^2^_101_=271.026, RMSEA=0.101 (90% CI 0.086-0.115), CFI=0.748, Akaike information criterion (AIC)=8667.727, and SRMR=0.086. In this model, there was a high correlation between factors 1 and 2 (*r*=0.997), but not between factors 1 and 3 (*r*=0.618) or factors 2 and 3 (*r*=0.591).

**Table 4:** Factor loadings (λ) for the 3-factor model with 16 items.

Item number	Item description	Factor	λ (SE)	*Z*-score
1	I frequently use the internet to gain health advice in the Diabetes Online Community.	1	1.00 (0.0)	999.0
2	I review multiple internet sources in the Diabetes Online Community before making a health decision for myself.	1	–0.494 (0.180)	2.741
3	I do not follow the health information that I find on social media in the Diabetes Online Community.^a^	1	0.951 (0.215)	4.412
4	I trust the health information that I find in the Diabetes Online Community.	1	1.127 (0.222)	5.083
5	I feel comfortable receiving health advice in the Diabetes Online Community.	1	1.531 (0.295)	5.184
6	I trust the health information that my friends on social media (Facebook, Twitter, Instagram, discussion forums) provide in the Diabetes Online Community.	1	1.503 (0.289)	5.203
7	I feel confident in my knowledge of the available online health resources in the Diabetes Online Community.	2	1.000 (0.0)	999.0
8	It is difficult for me to find health information online in the Diabetes Online Community.^a^	2	0.496 (0.180)	2.760
9	I feel confident in my ability to find accurate health information in the Diabetes Online Community.	2	0.803 (0.169)	4.737
10	When I am confronted with a health problem, I can usually find several solutions via advice in the Diabetes Online Community.	2	1.074 (0.150)	7.145
11	I prefer to get advice about medical devices (insulin pumps and CGMs^b^) from the Diabetes Online Community instead of my doctor.	2	0.783 (0.210)	3.729
12	When trying to understand my symptoms, my first resource is social media in the Diabetes Online Community.	2	1.143 (0.203)	5.625
13	I share health articles on my social media account (s) in the Diabetes Online Community.	3	1.00 (0.0)	999.0
14	I do not post health-related items on social media (Facebook, Twitter, Instagram, and/or discussion forums) in the Diabetes Online Community.^a^	3	1.093 (0.108)	10.144
15	I prefer to read the health information that I find on social media websites but not engage in online conversation about the health information in the Diabetes Online Community.^a^	3	0.730 (0.124)	5.904
16	I feel comfortable providing advice to others in the Diabetes Online Community.	3	0.583 (0.14)	5.098

^a^Item is reverse-coded owing to the negative phrasing.

^b^CGM: continuous glucose monitoring.

##### Two-Factor Model With 16 Items

The high correlation between factors 1 and 2 violated the discriminant validity of the measure. For this reason, factor 3 was removed from the list of items and we next evaluated the 2-factor model with CFA. Factor 1 is composed of items 1-12 and factor 2 is composed of items 13-16 (see Table 5 for factor loadings). The following indices presented a good model fit: χ^2^_103_=163.672, RMSEA=0.060 (90% CI 0.042-0.076), CFI=0.906, AIC=8631.384, and SRMR=0.072. In addition, the interfactor correlation between factors 1 and 2 was *r*=0.401.

**Table 5:** Factor loadings (λ) for the 2-factor model with 16 items.

Item number	Item description	Factor	λ (SE)	*Z*-score
1	I frequently use the internet to gain health advice in the Diabetes Online Community.	1	1.00 (0.0)	999.0
2	I review multiple internet sources in the Diabetes Online Community before making a health decision for myself.	1	0.499 (0.175)	2.848
3	I do not follow the health information that I find on social media in the Diabetes Online Community.^a^	1	0.917 (0.203)	4.514
4	I trust the health information that I find in the Diabetes Online Community.	1	1.087 (0.204)	5.337
5	I feel comfortable receiving health advice in the Diabetes Online Community.	1	1.457 (0.264)	5.515
6	I trust the health information that my friends on social media (Facebook, Twitter, Instagram, discussion forums) provide in the Diabetes Online Community.	1	1.440 (0.267)	5.40
7	I feel confident in my knowledge of the available online health resources in the Diabetes Online Community.	1	1.037 (0.184)	5.632
8	It is difficult for me to find health information online in the Diabetes Online Community.^a^	1	0.492 (0.186)	2.639
9	I feel confident in my ability to find accurate health information in the Diabetes Online Community.	1	0.851 (0.205)	4.159
10	When I am confronted with a health problem, I can usually find several solutions via advice in the Diabetes Online Community.	1	1.107 (0.161)	6.889
11	I prefer to get advice about medical devices (insulin pumps and CGMs^b^) from the Diabetes Online Community instead of my doctor.	1	0.845 (0.216)	3.912
12	When trying to understand my symptoms, my first resource is social media in the Diabetes Online Community.	1	1.187 (0.211)	5.631
13	I share health articles on my social media account (s) in the Diabetes Online Community.	2	1.105 (0.0)	999.0
14	I do not post health-related items on social media (Facebook, Twitter, Instagram, and/or discussion forums) in the Diabetes Online Community.^a^	2	1.00 (0.112)	9.885
15	I prefer to read the health information that I find on social media websites but not engage in online conversation about the health information in the Diabetes Online Community.^a^	2	0.728 (0.123)	5.914
16	I feel comfortable providing advice to others in the Diabetes Online Community.	2	0.578 (0.114)	5.086

^a^Item is reverse-coded owing to the negative phrasing.

^b^CGM: continuous glucose monitoring.

### Correlations

Importantly, several factors of diabetes distress were correlated with factors of the ATSHIO (Table 6): powerlessness and factor 1 (*r*=0.198, *P*=.01), hypoglycemia distress and factors 1 and 2 (*r*=0.153, *P*=.05 and *r*=0.158, *P*=.04, respectively), management distress and factor 2 (*r*=0.169, *P*=.03), physician distress and factor 1 (*r*=0.204, *P*=.008), and family distress and factor 2 (*r*=0.219, *P*=.005).

**Table 6:** Correlations of various scale items with Attitudes Toward Seeking Health Information Online factors for validation.

Scale items		Factor 1			Factor 2	
		*r*	*P* value	*r*		*P* value
**Diabetes distress**						
	Powerlessness	0.198	.01	NS^a^		—^b^
	Hypoglycemia distress	0.153	.05	0.158		.04
	Management distress	*NS*		0.169		.03
	Physician distress	0.204	.008	NS		—
	Friend/family distress	NS		0.219		.005
**Social provisions**						
	Attachment	0.183	.02	0.269		<.001
	Social integration	0.260	.001	0.276		<.001
	Reassurance of worth	0.251	.001	0.353		<.001
	Reliable alliance	0.273	<.001	0.264		<.001
	Guidance	0.341	<.001	0.314		<.001
	Opportunity for nurturance	0.172	<.001	0.324		<.001
eHealth literacy		0.413	<.001	0.197		.01
Hemoglobin A_1c_		NS	—	–0.358		<.001
Age		NS	—	–0.156		.04

^a^NS: not significant.

^b^Not applicable.

## Discussion

### Principal Findings

In this study, a scale examining online health information–seeking for individuals with T1D was developed and validated. This scale measures multiple types of peer-provided social support and examines how peers broker health information. Scale development was necessary due to the lack of existing scales addressing real-world experiences of seeking chronic disease–related information. We developed a reliable 2-factor, 16-item scale. Furthermore, this project examined the relationships between the measure of seeking health information online and the scale items of eHealth literacy, social provisions, and diabetes distress to establish validity by demonstrating the magnitude of these relationships.

Regarding CFA model comparison, the 2-factor, 16-item scale had small standardized residuals [32] and provided a good model fit. The majority of the project’s scales had excellent reliability, whereas a few scales used to validate the measure demonstrated adequate reliability, as indicated by the Cronbach α coefficient, including Social Provisions-Social Integration, Social Provisions-Reassurance of Worth, Diabetes Distress-Management Distress, Diabetes Distress-Eating Distress, and Attitudes Toward Seeking Health Information Online (factor 1), and fair reliability for Attitudes Toward Seeking Health Information Online (factor 2). The findings from this study will contribute to the knowledge base of the health care of adults with T1D. Participants were forthcoming about the items of the scale and provided recommendations, as they are a very active and communicative population in the context of social media.

As expected, both factors were positively related to eHealth literacy. Additionally, the Trusting and Evaluating Online Health Information factor was positively related to the Social Provisions factors (Attachment, Social Integration, Reassurance of Worth, Reliable Alliance, Guidance, and Opportunity). Thus, this study extends what is known about informational support, as a type of social support, in the context of online health information–seeking. The factor Engaging with Online Health Information in the DOC was also found to be positively related to several Social Provisions items (Attachment, Social Integration, Reassurance of Worth, Reliable Alliance, Guidance, and Opportunity for Nurturance). These relationships are to be expected, as informational support is a type of social support.

### Diabetes Distress

Of interest, Trusting and Evaluating Online Health Information (factor 1) was positively related to multiple types of Diabetes Distress items (Powerlessness, Hypoglycemia Distress, Physician Distress). These findings are a unique contribution to the T1D literature because they provide support that key diabetes-related constructs impacting health behaviors also impact health information–seeking. These findings are significant because, to the best of our knowledge, this study is the first to assess these relationships. These findings are a unique contribution to the T1D literature because they provide support that with more feelings of distress toward managing T1D, hypoglycemia-related distress, and diabetes-related distress related to friends and family, individuals are engaging more with online health information in the DOC. With more diabetes-related distress comes more engagement in the DOC and more trust in the information found online.

### Clinical Implications

This study highlights that with more distress toward managing T1D, hypoglycemia-related distress, and diabetes-related distress related to friends and family, people with T1D are engaging more with online health information in the DOC. This is important because instead of seeking support from their health care team, they are seeking support from the DOC (which is available 24 hours a day, 7 days a week). Clinicians may be able to use this scale as a starting point for a discussion with their patients with T1D about how they seek information online and how their clinicians can better support them when they need information quickly. This is especially poignant for the current generation of clinicians who are using telehealth.

### Limitations and Future Directions

Although this study provides an innovative, valid, and reliable scale, there are a few important limitations from which future research may build upon. The sample mostly comprised female participants of White race who were well-educated. Future research in this area should also seek to collect data from minority populations because much of the existing DOC research does not represent the diversity that exists in the online community. Similar research considering and incorporating caregivers for adolescents with T1D would be beneficial because these individuals also engage in the DOC. The developed ATSHIO scale was created for the T1D community but could be tailored for other chronic disease groups who seek health information online.

Future research should be performed based on the feedback provided in this study for the ATSHIO scale to further confirm the findings, further validate its factor structure, and establish reliability of those factors. Future research should also aim to increase the reliability of both factors of the ATSHIO scale. Due to the nature of potential biases inherent to self-reported data, future research should seek to incorporate other sources of data beyond self-reported data, including electronic medical record data.

### Conclusions

These findings provide support for the relationships between ATSHIO, social provisions, diabetes distress, and T1D-related health outcomes and behaviors. With a better understanding of the roles of online social support and seeking health information online on disease management, this project serves as the first of several series of studies to improve use of the DOC and facilitate constructions of interventions that encourage or discourage specific aspects of each behavior. From these results, clinicians may encourage people with diabetes to seek social and informational support online. People with diabetes should be educated on health literacy to safely navigate the diabetes online community.

## Abbreviations

AIC: Akaike information criterion
ATSHIO: Attitudes Toward Seeking Health Information Online
CFA: confirmatory factor analysis
CFI: comparative fit index
DOC: Diabetes Online Community
RMSEA: root mean square error of approximation
SRMR: standardized root mean square residual
T1D: type 1 diabetes
TLI: Tucker-Lewis index
